# Necrotic-like BV-2 microglial cell death due to methylmercury exposure

**DOI:** 10.3389/fphar.2022.1003663

**Published:** 2022-11-02

**Authors:** B. Martins, J. P. Novo, É. Fonseca, R. Raposo, V. A. Sardão, F. Pereira, R. B. Oriá, C. Fontes-Ribeiro, J. Malva

**Affiliations:** ^1^ Coimbra Institute for Clinical and Biomedical Research (iCBR), University of Coimbra, Coimbra, Portugal; ^2^ Institute of Pharmacology and Experimental Therapeutics, Faculty of Medicine, University of Coimbra, Coimbra, Portugal; ^3^ Center for Innovative Biomedicine and Biotechnology (CIBB), University of Coimbra, Coimbra, Portugal; ^4^ Experimental Biology Core, Health Sciences Center, University of Fortaleza, Fortaleza, Brazil; ^5^ Center for Neuroscience and Cell Biology (CNC), UC Biotech, University of Coimbra, Cantanhede, Portugal; ^6^ Laboratory of Tissue Healing, Ontogeny, and Nutrition, Department of Morphology and Institute of Biomedicine, School of Medicine, Federal University of Ceará, Fortaleza, Brazil

**Keywords:** methylmercury (MeHg), neuroinflammation, neurotoxicity, neurodegeneration, microglial cells

## Abstract

Methylmercury (MeHg) is a dangerous environmental contaminant with strong bioaccumulation in the food chain and neurotoxic properties. In the nervous system, MeHg may cause neurodevelopment impairment and potentially interfere with immune response, compromising proper control of neuroinflammation and aggravating neurodegeneration. Human populations are exposed to environmental contamination with MeHg, especially in areas with strong mining or industrial activity, raising public health concerns. Taking this into consideration, this work aims to clarify pathways leading to acute toxic effects caused by MeHg exposure in microglial cells. BV-2 mouse microglial cells were incubated with MeHg at different concentrations (0.01, 0.1, 1 and 10 µM) for 1 h prior to continuous Lipopolysaccharide (LPS, 0.5 μg/ml) exposure for 6 or 24 h. After cell exposure, reactive oxygen species (ROS), IL-6 and TNF-α cytokines production, inducible nitric oxide synthase (iNOS) expression, nitric oxide (NO) release, metabolic activity, propidium iodide (PI) uptake, caspase-3 and -9 activities and phagocytic activity were assessed. MeHg 10 µM decreased ROS formation, the production and secretion of pro-inflammatory cytokines IL-6, TNF-α, iNOS immunoreactivity, the release of NO in BV-2 cells. Furthermore, MeHg 10 µM decreased the metabolic activity of BV-2 and increased the number of PI-positive cells (necrotic-like cell death) when compared to the respective control group. Besides, MeHg did not interfere with caspase activity or the phagocytic profile of cells. The short-term effects of a high concentration of MeHg on BV-2 microglial cells lead to impaired production of several pro-inflammatory mediators, as well as a higher microglial cell death *via* necrosis, compromising their neuroinflammatory response. Clarifying the mechanisms underlying MeHg-induced neurotoxicity and neurodegeneration in brain cells is relevant to better understand acute and long-term chronic neuroinflammatory responses following MeHg exposure.

## Introduction

Methylmercury (MeHg) is a widespread environmental pollutant, of utmost public concern, due to its marked neurotoxicity and potential long-term deleterious effects on human health (Clarkson et al., 2003). The developmental neurotoxicity in humans has been firstly reported in 1956 in Minamata city after poisoning catastrophes (Harada, 1995). Importantly, the neurotoxicological effects of MeHg poisoning were reported in several populations worldwide, potentially associated with serious motor function and extensive brain impairment (Bakir et al., 1973; Ceccatelli et al., 2010). The symptoms of MeHg poisoning include impairment of gait and speech, ataxia, muscle weakness, tremor, abnormal eye movement, weight loss, memory, and hearing loss, accompanied by the disruption and shrinkage of neuronal cells. Further, whereas the number of acute cases has been decreasing over time, the number of chronic patients and children presenting several symptoms has been raising. Surprisingly, children born from asymptomatic women or with minimal symptoms, also displayed severe neurological impairment, highlighting the vulnerability of the developing nervous system (Bakir et al., 1973; Choi et al., 1983; Eto, 1997; Clarkson et al., 2003).

Mercury can be released into the environment through natural or anthropogenic sources, such as volcanic eruptions and gold mining, respectively. In nature, MeHg is generated through the biomethylation of inorganic mercury in aquatic environments by sulfate-reducing bacteria (Novo et al., 2021). Once in the food chain, MeHg is bioaccumulated and biomagnified, reaching high concentrations in fish and seafood. Subsequently, populations subsisting on seafood for their diet are potentially exposed to chronic high levels of MeHg, making these regular consumers susceptible to more severe toxicity (Ni et al., 2010; Antunes dos Santos et al., 2016). Approximately 95% of MeHg ingested is absorbed within the gastrointestinal tract, transported into the bloodstream, and slowly redistributed into different organs. Due to its affinity to thiol groups, MeHg conjugates with sulfhydryl-containing molecules such as albumin, hemoglobin, or L-cysteine which increases its water solubility, facilitating the circulatory distribution of MeHg (Tian et al., 2016). The L-cysteine-MeHg complex can diffuse to all brain areas, crossing the blood-brain barrier through the neutral amino acid transport system (LAT1) (Yin et al., 2008). However, MeHg is not equally distributed in the different brain cell types, being found in lower concentrations in neurons and preferentially accumulated in certain glial cells, such as astrocytes and microglia (Charleston et al., 1994). Therefore, long-term exposure to MeHg, due to the consumption of contaminated fish, has been found to induce several neurotoxic effects, compromising human health with concentrations going from 2.5 to 10 μM. MeHg toxicity has been identified as the second-most prevalent cause of acute heavy metal poisoning (Clarkson et al., 2003; Pinheiro et al., 2007; Kim et al., 2020).

Besides the previously mentioned symptoms associated with MeHg poisoning, several studies have reported the neurotoxicity effect of MeHg, at cellular levels, in the central nervous system (CNS) (Castoldi et al., 2000; Eskes et al., 2002; Ni et al., 2010). It has been reported that MeHg-induced neurotoxicity is associated with excessive reactive oxygen species (ROS) generation. ROS can exert direct cell damage, through several mechanisms, such as DNA damage, lipid peroxidation, protein oxidation, perturbing protein function and activity, disturbing cellular antioxidant defense, and leading to further ROS accumulation (Castoldi et al., 2000; Shenker et al., 2002). Mitochondrial functions also seem to be impaired upon MeHg exposure by decreasing ATP content and reduced mitochondrial membrane potential, leading to mtDNA mutations and subsequently inducing apoptotic cell death (Wang et al., 2016). Furthermore, MeHg stimulates the microglial production and secretion of pro-inflammatory cytokines and chemokines, exacerbating neuroinflammation. For instance, MeHg-exposed microglia have been found to enhance the production and release level of IL-6 (Eskes et al., 2002). Evidence suggests that accumulated impairment in brain cells caused by MeHg can induce apoptosis at low concentrations, while at higher concentrations MeHg mainly induces necrosis (Eskes et al., 2002; Wang et al., 2016). Therefore, the type of MeHg-induced cell death seems to be correlated with the time and concentration of the contaminant exposure, modulating the neuroinflammatory and neurotoxic outcome.

Microglia are a specialized population of macrophage-like cells, activated by a broad range of stimuli, promptly responding to pathogen-associated (PAMP) and damage-associated molecular patterns (DAMP) that may trigger a complex array of neuroinflammatory responses in the CNS (Venegas and Heneka, 2017). Microglia perform a multitude of important functions, by inducing neuronal differentiation (Göritz et al., 2007), phagocytosis of cellular debris (Hanisch and Kettenmann, 2007), releasing a variety of cell signaling factors (Bessis et al., 2007), and mediating immune responses (Griffiths et al., 2009). Those cells constantly survey the brain milieu and in response to altered microenvironment homeostasis, microglia cells become reactive and can shift their morphology, which facilitates migration to the injury site (Thameem Dheen et al., 2007; Griffiths et al., 2009). Marked and disseminated microglial reaction may result and perpetuate the neuroinflammatory state, also contributing to the neurodegeneration through the release of free radicals, excessive nitric oxide (NO), inducible nitric oxide synthase (iNOS), and multiple chemokines and cytokines, such as IL-1β, IL-6, IL-8, IL-12, IL-15, IL-10, and TNF-α (Shinozaki et al., 2019; Cheng et al., 2021).

Microglia are thought to have an essential role in the neurogenic niche of the adult hippocampus. Those cells have not only the capacity to modulate hippocampal neurogenesis by removing cells but also can influence the proliferation, differentiation process into neurons or glia, and survival of newborn cells (Gemma and Bachstetter, 2013). Previous reports suggest that under MeHg brain cells intoxication the neurogenesis process is compromised. The MeHg-induced neuroinflammation (Tasneem et al., 2016) impacts the hippocampal structure and function (Sokolowski et al., 2013). Moreover, early exposure in older juveniles to MeHg affects later neurogenesis and MeHg sensitivity can decrease with age (Obiorah et al., 2015). Thus, it is thought that adult hippocampal neurogenesis may be modulated by environmental factors that also affect microglia, thus suggesting that there may be a connection between microglial cells, neurogenesis, cognitive function, and environment quality (Valero et al., 2016; Raposo et al., 2020).

In spite of the extensive characterization of neurotoxicity caused by MeHg exposure, the acute toxic effects caused by MeHg exposure in microglial cells are poorly understood. Therefore, in the present study, we aimed to clarify pathways leading to acute toxicity caused by MeHg exposure in microglial cells and molecular/cellular pathways leading to cell death.

## Materials and methods

### Cell culture and methylmercury treatment

BV-2 immortalized mouse microglial cell line (ICLC, Genova, Italy) was maintained in Roswell Park Memorial Institute (RPMI) medium (GIBCO, Invitrogen, Carlsbad, CA) supplemented with 10% heat-inactivated foetal bovine serum (FBS) (GIBCO, Invitrogen), 2 mM L-glutamine (GIBCO, Invitrogen), and antibiotics (100 U/ml penicillin, 100 μg/ml streptomycin, GIBCO, Invitrogen) at 37°C in a humidified atmosphere of 5% CO_2_. Twenty-4 hours after seeding, BV-2 cells were incubated with different MeHg concentrations (0.01, 0.1, 1 and 10 μM) for 1 h and then challenged continuously with 0.5 μg/ml Lipopolysaccharide (LPS, O111:B4, Calbiochem) for 6 h or 24 h. This protocol has been applied for the following methods: Quantification of ROS; Enzyme-linked Immunosorbent Assay (ELISA); Resazurin Reduction Assay; Propidium Iodide (PI) staining; Measurement of Caspase activity assay; Immunocytochemistry and Phagocytic activity assay; Western Blot and Griess Assay.

### Quantification of ROS

Intracellular ROS production was evaluated using a 2′,7′-dichlorodihydrofluorescein diacetate (H_2_DCFDA) probe (Thermo Fisher, Waltham, United States). BV-2 microglial cells were seeded in a 96-well plate at a density of 6.25 x 10^4^ cells/cm^2^. After treatments, cells were incubated with 5 μM H2DCFDA freshly prepared in KREBS solution (142 mM NaCl; 4 mM KCl; 1 mM MgCl_2_; 1 mM CaCl_2_; 10 mM glucose; 10 mM HEPES) for 1 h. Fluorescence was measured (excitation/emission: 485/528 nm) using a microplate reader (Biotek Synergy HT, Winooski, United States).

### ELISA assay to determine TNF-α and IL-6

Cells were seeded at a density of 2.5 × 10^5^ cells/cm^2^ and treated as previously described. For the quantification of intracellular and extracellular cytokines protein levels, IL-6 and TNF-α uncoated ELISA kits (Invitrogen, Thermo Fisher) were used following the manufacturer’s instructions. Briefly, after treatments of cells, the supernatants were centrifuged at 12,000 rpm and stored at −80°C. Cells were washed with PBS and left at room temperature (RT) in cell extraction buffer (Thermo Fischer, FNN0011) supplemented with protease inhibitor solution (50 μl/ml Protease Inhibitor Cocktail, Sigma, and 1 mM PMSF). Protein concentration was determined by the Bicinchoninic acid (BCA) method and stored at −80°C until further use. Microtiter plates (MaxiSorp, Nunc, and Thermo Fisher) were coated with capture antibody in coating buffer. Plates were sealed and left overnight at 4°C. Wells were washed with PBS containing 0.05% Tween-20, blocked with 1X assay buffer, and left at RT for 1 h. After washing, 100 μL of each sample was added as well as standard solutions after performing 2-fold serial dilutions of the top standard. The plates were sealed and left incubating overnight at 4°C. Afterwards, a detection antibody was added, and the plates were sealed and incubated at RT for 1 h under agitation. Washes were repeated and Streptavidin-HRP was added to each well. Then, the substrate was added to each well and incubated at RT for 15 min. Finally, 50 μL/well of stop solution (2 M H_2_SO_4_) was added. Optical density was recorded at 450 nm and 570 nm (the values corresponding to 570 nm were subtracted from those obtained with 450 nm) in a microplate reader (Biotek Synergy HT, Winooski, United States).

### Western Blot

BV-2 microglial cells were seeded in a 12-well plate at a density of 2.5 × 10^5^ cells/cm^2^. After appropriate treatment, cells were scrapped, homogenized, and lysed using a commercial cell extraction buffer (Thermo Fischer) supplemented with protease inhibitor solution (50 μl/ml Protease Inhibitor Cocktail (Sigma) and 1 mM PMSF). Protein concentration was determined by the BCA method and samples were stored at −80°C until further use. 50 μg of total protein was separated by electrophoresis on 7.5% polyacrylamide gel electrophoresis, transferred onto nitrocellulose membrane (Millipore, Madrid, Spain), and blocked by 5% non-fat dry milk (NFDM) in Tris-buffer saline solution with tween-20 (TBS-T) for 1 h at RT. Then, the membranes were probed with primary antibody, iNOS (D6B6S) Rabbit mAb (#13120S, 1:1,000 Cell Signaling) in 5% NFDM diluted in 1 X TBST-T overnight at 4°C. Membranes were then washed and incubated with HRP-conjugated secondary antibodies for 1 h at RT (Anti-rabbit IgG HRP-linked, #7074, 1:10,000, Cell Signaling and Anti-mouse IgG HRP-linked, #7076, 1:10,000, Cell Signaling). For endogenous control immunolabelling, immunoblots were reprobed with mouse monoclonal anti-GAPDH antibody (#CB1001, 1:5,000, Merck Millipore) to ensure equal sample loading. Protein immunoreactive bands were visualized in a ChemiDoc Imaging System (Biorad Laboratories, CA, United States) after incubation of the membrane with Clarity Western ECL reagent (Biorad) and densitometric analysis were performed using ImageLab software.

### Nitric oxide determination

The production of NO was assessed indirectly by measuring nitrites released into the culture media. BV-2 microglial cells were seeded in 96-well plates at a density of 6.25 × 10^5^ cells/cm^2^. After treatments, the supernatants were collected and NO production was determined using a NO detection kit (Promega, Madison, EUA), according to the manufacturer’s instructions. Briefly, 50 μl of supernatant was incubated with an equal volume of sulfanilamide solution for 10 min and then with (1-Naphthyl)-ethylenediamine (NED) Solution for an additional 10 min. The absorbance of the reaction mixtures was measured at 520 nm using a microplate reader (Biotek Synergy HT, Winooski, United States).

### Resazurin reduction assay

The metabolic status of BV-2 cultures was evaluated using a resazurin reduction assay. Cells were seeded in 24-well plates at a density of 2.5 × 10^5^ cells/cm^2^. After treatments, cells were incubated with a 0.5 mg/ml resazurin solution prepared in culture medium for 1 h at 37°C. Absorbance was measured at 570 nm and 620 nm with a microplate reader (Biotek Synergy HT, Winooski, United States). The absorbance values obtained at 620 nm were subtracted from those at 570 nm.

### PI staining

Necrotic-like cell death was assessed by PI staining. BV-2 microglial cells were seeded in a 12-well plate at a density of 2.6 × 10^3^ cells/cm^2^. After treatments, cells were incubated with 10 μg/ml of freshly prepared PI (Sigma) for 15 min at 37°C. Then, cells were fixed with 4% paraformaldehyde (PFA) containing 4% sucrose with Hoechst 33342 (#H1399,1:2000, Invitrogen) for 15 min at 37°C. Cells were imaged under an inverted Axiovert 200 M fluorescence microscope controlled by Axiovision software. For each experimental condition, a minimum of 4 fields were randomly acquired under ×50 magnification. Fiji software was used to count cells and the percentage of PI-positive cells was calculated.

### Immunocytochemistry

For immunocytochemistry, coverslips were placed in 12-well plates and coated with poly-D-Lysine and Laminin. Next, cells were seeded at a density of 5 × 10^4^ cells/cm^2^. After the experiment, cells were fixed by incubating at RT with 4% PFA (Sigma) for 20 min and washed twice with PBS. The cells were then permeabilized for 5 min in PBS containing 1% Triton X-100 (Sigma), and subsequently incubated with 3% BSA, for 10 min to block the unspecific binding of the antibodies. Following the blocking step, the cells were incubated with the primary antibody iNOS (D6B6S) Rabbit mAb (#13120S, 1:200, Cell Signalling), diluted in 3% BSA, and incubated overnight at 4°C. Primary antibodies were revealed by appropriate secondary antibodies labelled with Alexa Fluor (Chicken anti-Rabbit IgG (H + L), Alexa Fluor™ 594, #A-21442, 1:250, Invitrogen) diluted in 3% BSA. Phalloidin (Flash Phalloidin™ Green 488, #424201, 1:200, Biolegend) and Hoechst 33342 (#H1399, 1:2000, Invitrogen) were added in 3% BSA for 5 min at RT, to visualize F-actin and to label nuclei, respectively. Finally, the stained cells were washed three times with PBS and mounted on slides using Dakocytomation fluorescent medium (Dakocytomation Inc, California, United States). Results were analyzed on a fluorescence microscope (Axio Observer. Z1/7 Inverted Microscope, Carl Zeiss, Germany). For each experimental condition, a minimum of 5 fields were randomly acquired under ×400 magnification.

### Caspase activity assay

Caspase-3 and caspase-9 activity were determined by a caspase colorimetric activity assay, as previously described by Moreira and co-workers (Moreira et al., 2014). BV-2 cells were seeded in a 6-well plate at a density of 2.1 × 10^4^ cells/cm^2^ and incubated with different MeHg concentrations for 1 h and challenged with LPS for 6 h. After treatment cells were harvested after being scrapped, and resuspended in lysis buffer [50 mM HEPES, 100 mM NaCl, 0.1% CHAPS, 1 mM 1,4-dithiothreitol (DTT)], and incubated on ice for 15 min. The cell lysates were used to quantify the amount of protein using the BCA protein assay.

To test the protease activity of caspase-3 and caspase-9, 25 μg and 50 μg of protein from the respective sample extracts to assess were incubated with the assay buffer (50 mM HEPES, 100 mM NaCl, 2 mM EDTA, 1.2% CHAPS, 10% glycerol, 10 mM DTT) and the respective caspase substrates conjugated with chromophore molecule p-nitroanaline (pNA). These substrates were incubated at 37°C for 2 h with each sample, allowing the cleavage of p-NA by the caspase activity. After incubation, samples were quantified with a spectrophotometer (Cytation3 imaging reader, BioTek, United States) at a wavelength of 405 nm, through the colorimetric detection of the pNA. The method was calibrated with known concentrations of pNA, and the results were expressed as a concentration of pNA released (nm)/ug protein.

### Phagocytic activity assay

Phagocytic activity was evaluated with fluorescent latex beads (1 µm diameter, Sigma), as previously described with minor alterations (Aires et al., 2019). Thus, microglial cells were seeded in a 12-well plate at a density of 2.6 × 10^3^ cells/cm^2^ and incubated with fluorescent latex beads (0.00025% of final volume) for 1 h at 37°C before the end of timepoints. After incubation, cells were fixed with 4% PFA containing 4% sucrose, for 10 min, and stained with Phalloidin (Flash Phalloidin™ Red 594, #424203, 1:200, Biolegend) and Hoechst 33342 (#H1399, 1:2000, Invitrogen), in 0.025% Triton x-100 for 1 h at RT. Cell preparations were mounted in Dakocytomation fluorescent medium and sealed with nail polish.

The preparations were observed using an inverted Axiovert 200 M fluorescence microscope controlled by Axiovision software. For each experimental condition, a minimum of 5 fields were randomly acquired under ×200 magnification.

The phagocytic efficiency was determined using the following formula:

$$Phagocytic\;Efficiency\left(\%\right)=\left[\;\frac{\left(1\times X1+2\times X2+3\times X3\ldots +n\times Xn\right)}{Total\;number\;of\;cells}\right]\times 100$$

Where Xn represents the number of cells containing n beads (*n* = 1, 2, … , up to a maximum of 6). For cells containing more than 5 internalized beads, n was always considered as 6. Fiji software was used to count cells and beads.

### Data analysis

Statistical analysis was performed using GraphPad Prism 6.0 (GraphPad Software, United States). Statistic tests and the number of experiments are discriminated in the respective figure legends. Statistical significance was considered relevant for *p* values <0.05 using a one-way ANOVA analysis of variance followed by Bonferroni’s post-hoc. Data were presented as the mean ± standard error of the mean (SEM).

Every experimental condition was tested in three sets of independent experiments unless otherwise stated.

## Results

### MeHg decreased ROS production on LPS-treated BV-2 microglial cells

**FIGURE 1:**
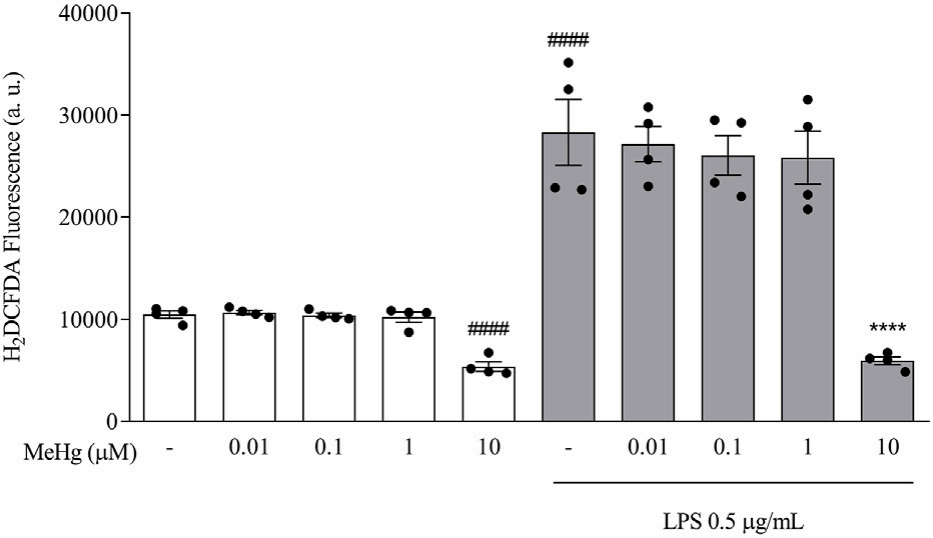
Effects of MeHg exposure (0.01, 0.1, 1, and 10 µM) on ROS production using the fluorescent probe H_2_DCFDA. Cells were pre-exposed to MeHg for 1h, followed by LPS 0.5 μg/ml for additional 6 h ####*p* < 0.0001 vs. Control ****p < 0.0001 vs. LPS. Data are expressed as Mean ± SEM, *n* = 4. One-way ANOVA, followed by Bonferroni’s multiple comparisons test.

To investigate the impact of MeHg on oxidative stress, intracellular ROS levels were measured in the different BV-2 cell groups treated with MeHg for 1 h and subsequently stimulated with LPS for 6 h. MeHg at 10 μM concentration reduced the fluorescence intensity, from 10,451.5 ± 720.4 a. u. (control group) to 5370.3 ± 920.0 (10 µM-MeHg, *p* < 0.0001, Figure 1). Following exposure to LPS 0.5 μg/ml, a significant increase in H_2_DCFDA fluorescence was observed (28,307.5 ± 6,471.0 a. u, *p* < 0.0001), compared to non-treated cells. Additionally, under LPS treatment, a significant reduction of ROS production was detected on cells challenged with 10 µM-MeHg (5,928.5 ± 783.8 a. u, *p* < 0.0001), when compared to the respective control group (28,307.5 ± 6,471.0 a. u.). Our results point out that MeHg 1 μM, or lower concentrations, did not significantly affect LPS-induced ROS production (*p* > 0.05). These results indicate that a higher concentration (10 µM) of MeHg reduces ROS production by microglia.

### MeHg reduced the production and release of pro-inflammatory cytokines on LPS-treated BV-2 microglial cells

**FIGURE 2:**
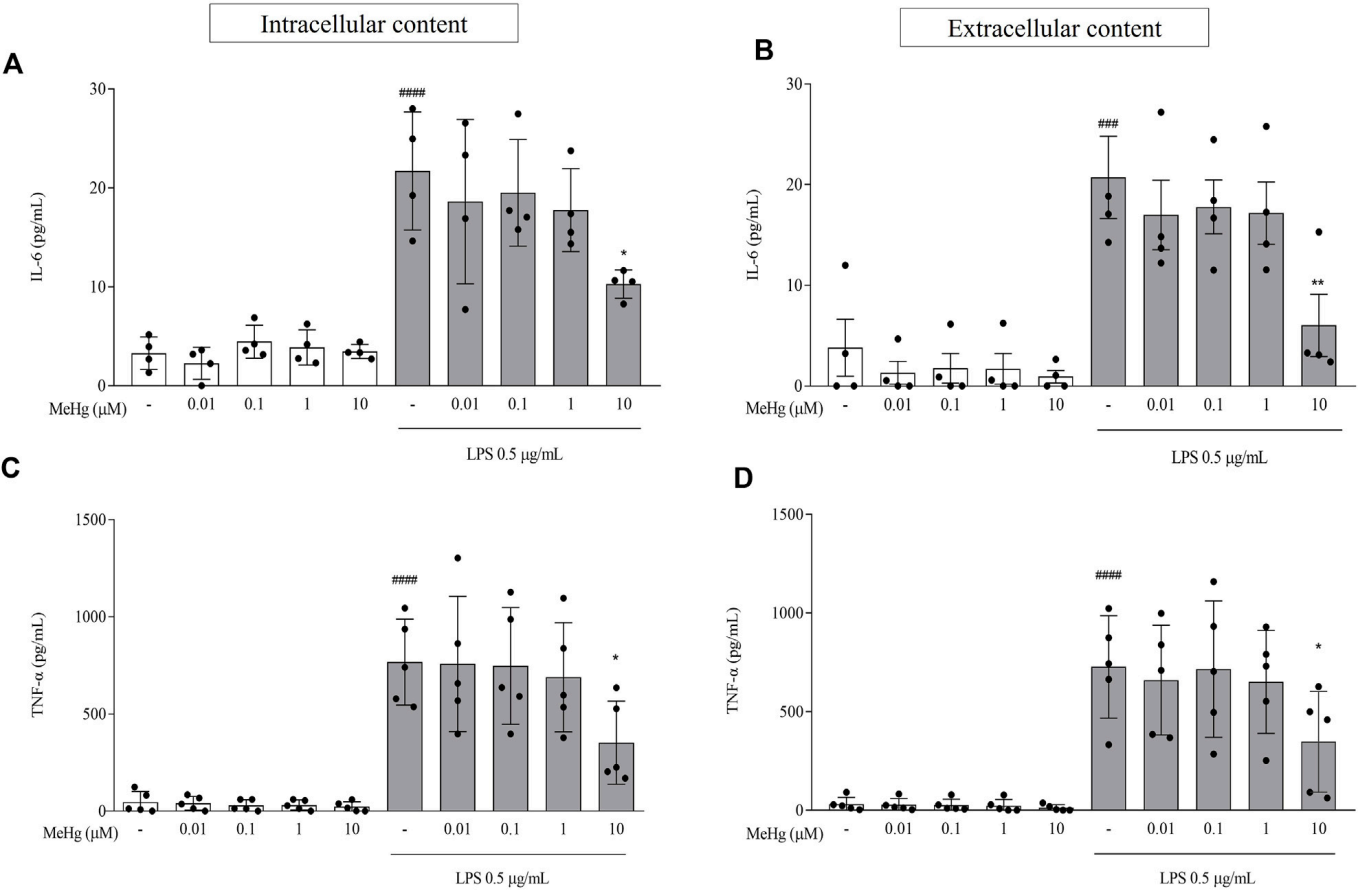
Effects of MeHg exposure (0.01, 0.1, 1, and 10 µM), on IL-6 and TNF-α contents using ELISA assay. **(A)** IL-6 and **(C)** TNF-α intracellular contents and **(B)** IL-6 and **(D)** TNF-α extracellular contents were quantified after exposure to different MeHg concentrations on BV-2 microglial cells treated for 1 h and subsequently stimulated with LPS for 6 h ###*p* < 0.001 and ####*p* < 0.0001 vs. Control; *p < 0.05 vs. LPS. Data are expressed as Mean ± SEM. **(A)** and **(C)** *n* = 4 and **(B)** and **(D)** *n* = 5. One-way ANOVA, followed by Bonferroni’s multiple comparisons test.

To determine the MeHg effect on microglial pro-inflammatory cytokine production and release, cells were treated with MeHg for 1 h and subsequently challenged with LPS for 6 h. IL-6 and TNF-α were determined by ELISA. In the absence of LPS, MeHg-treated cell cultures produced and released low levels of IL-6 and TNF-α, that were not significantly different from control condition (*p* > 0.05; Figure 2). However, both intracellular and extracellular levels of IL-6 and TNF-α, rose abruptly in response to LPS stimulation, when compared to the control (*p* < 0.0001 for intracellular levels of IL-6 and TNF-α and extracellular contents for TNF-α; *p* < 0.001 for extracellular of IL-6). Moreover, upon pre-exposure to 10 µM MeHg cells stimulated with LPS exhibit a significant decrease in intracellular contents of both IL-6 and TNF-α, from 21.7 ± 6.0 pg/ml and 767.3 ± 221.1 pg/ml to 10.3 ± 1.4 pg/ml (*p* < 0.05) and 352.1 ± 213.6 pg/ml (*p* < 0.05), respectively, when compared to the respective control groups. We also observed a parallel decrease in 10 µM MeHg cells challenged with LPS on extracellular content of IL-6 and TNF-α in the same conditions going from 20.7 ± 8.2 pg/ml and 727.0 ± 259.5 pg/ml to 6.0 ± 6.2 pg/ml (*p* < 0.05) and 347.6 ± 255.2 pg/ml (*p* < 0.05), respectively. Furthermore, lower concentrations of MeHg did not affect IL-6 and TNF-α program under LPS exposure (*p* > 0.05). These results suggest that MeHg at high concentrations impairs the microglial content and release of pro-inflammatory cytokines.

### MeHg impaired LPS-induced iNOS and NO levels

**FIGURE 3:**
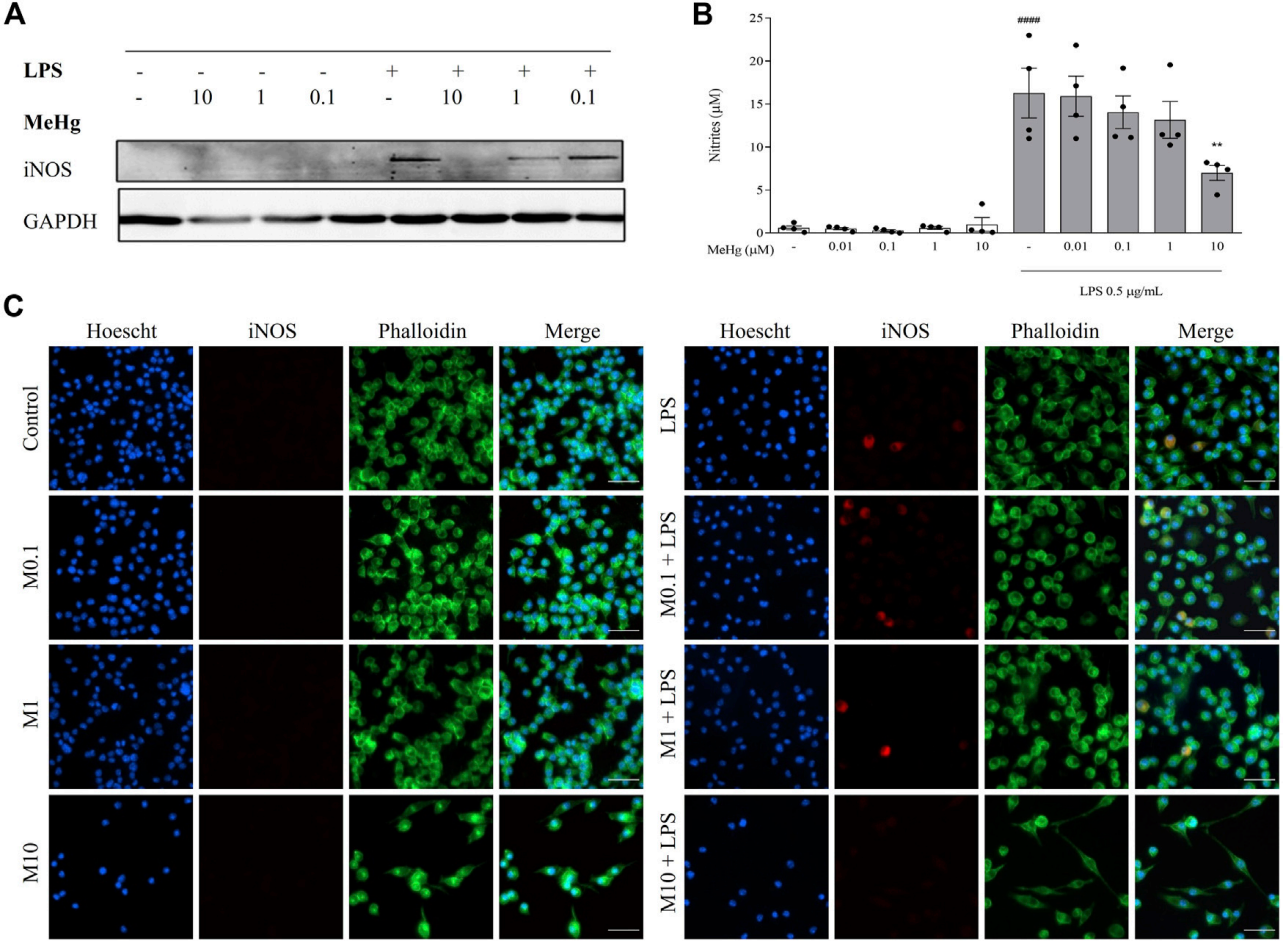
Effect of MeHg exposure (0.01, 0.1, 1 and 10 µM) on iNOS expression and nitrites production. **(A)** iNOS density and **(B)** nitrates levels were quantified after exposure to different MeHg concentrations on BV-2 microglial cell treated for 1 h subsequently stimulated with LPS for 24 h. GAPDH was used as the loading control. **(C)** Representative images of Hoechst 33342 (blue), iNOS (red) and Phalloidin (green) labelled cells, obtained through immunocytochemistry. Scale bar, 50 μm ####*p* < 0.0001 vs. Control; **p < 0.01 vs. LPS. Data are expressed as Mean ± SEM, *n* = 4. One-way ANOVA followed by Bonferroni’s multiple comparisons test.

To study the effect of MeHg on microglia NO metabolism, iNOS and nitrites (NO metabolites) levels were evaluated. The expression of iNOS was undetectable on control cells or cells treated with MeHg but was visibly induced by LPS treatment (Figure 3). Stimulation with LPS under the highest MeHg concentration resulted in a significant decrease of iNOS levels. Accordingly, LPS induced a significant increase in nitrites release (5.8 ± 1.7 µM, *p* < 0.0001), when compared to the control group (16.3 ± 7.0 µM). Furthermore, cells exposed to 10 µM-MeHg and subsequently challenged with LPS showed a significant decrease in nitrites release (7.0 ± 1.7 µM, *p* < 0.001), when compared to LPS-only treated cells (16.3 ± 5.8 µM). Lower concentrations of MeHg did not affect NO metabolism under LPS exposure (*p* > 0.05). This indicates that high concentrations of MeHg interfere with NO metabolism of microglial cells.

### MeHg impaired BV-2 metabolic activity and induced necrotic cell death

**FIGURE 4:**
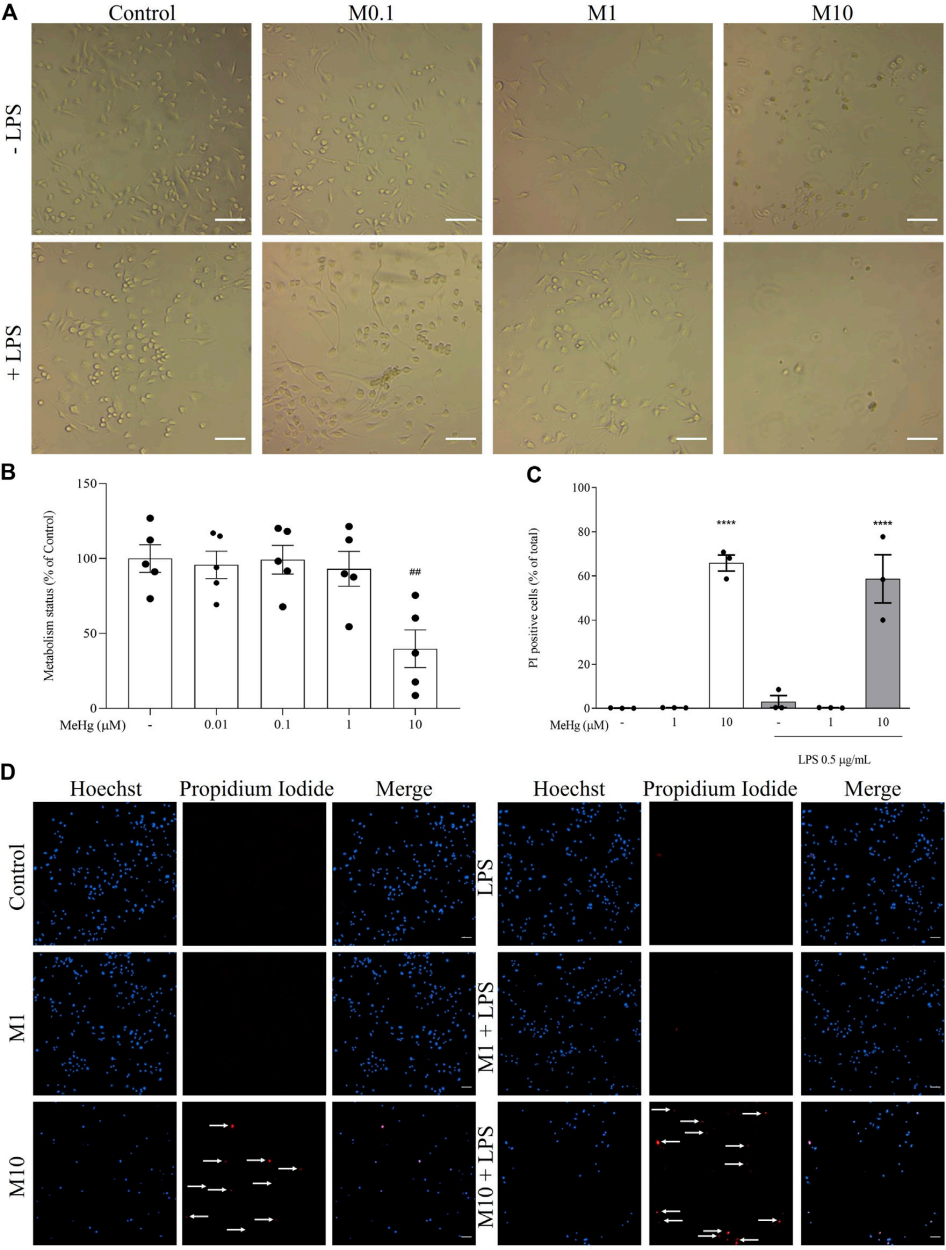
Effect of MeHg exposure (0.01, 0.1, 1, and 10 µM) on the metabolic activity and cell death by necrosis. **(A)** Representative images of cellular viability. Scale bars, 100 µm. **(B)** Assessment of the metabolism status of cells and **(C)** quantification of PI-positive cells after exposure to different MeHg concentrations on BV-2 microglial cell treated for 1 h subsequently stimulated with LPS for 6 h. **(D)** Representative images of Hoechst 33342 (blue) and PI-positive (red) labelled cells. Arrows show PI-positive cells. Scale bar, 200 μm ##*p* < 0.01 and ####*p* < 0.0001 vs. Control; ****p < 0.0001 vs. LPS. Data are expressed as Mean ± SEM, *n* = 3. One-way ANOVA, followed by Bonferroni’s multiple comparisons test.

To investigate the effects of MeHg concentration-related toxicity on microglia, metabolic activity was investigated after the exposure to MeHg. Upon 6 h MeHg exposure, cell metabolic activity decreased significantly in cells exposed to 10 µM MeHg (39.8 ± 22.5%, *p* < 0.001), comparing to control (100 ± 20.5%, Figures 4A,B).

Based on these results, 10 and 1 μM MeHg were selected to detect the presence of necrotic cell death by PI assay. Cells incubated with 10 µM of MeHg and stimulated or not with LPS (65.8 ± 4.8 and 58.7 ± 12.7%, respectively, *p* < 0.0001) were found to significantly enhance necrotic-like features compared to their respective controls (0.1 ± 0.09 and 2.3 ± 3.1%, *p* < 0.0001, Figures 4C,D). Thus, these results reveal that necrosis is the predominant form of cell death induced by micromolar MeHg concentrations.

### MeHg does not interfere with caspase activity

To test whether MeHg-induced microglial cell death also involves apoptotic routes, caspase-3 and caspase-9 activities were assessed. Colorimetric activity assay analyses did not show statistical differences in both cleaved caspase-3 and -9 levels in MeHg-treated cell cultures (0.1, 1, and 10 µM) for 6 h compared to their respective control cells (*p* > 0.05) (data not shown). This indicates that MeHg induces microglial cell death independently of caspases pathways.

### MeHg did not interfere with LPS-induced phagocytic profile

**FIGURE 5:**
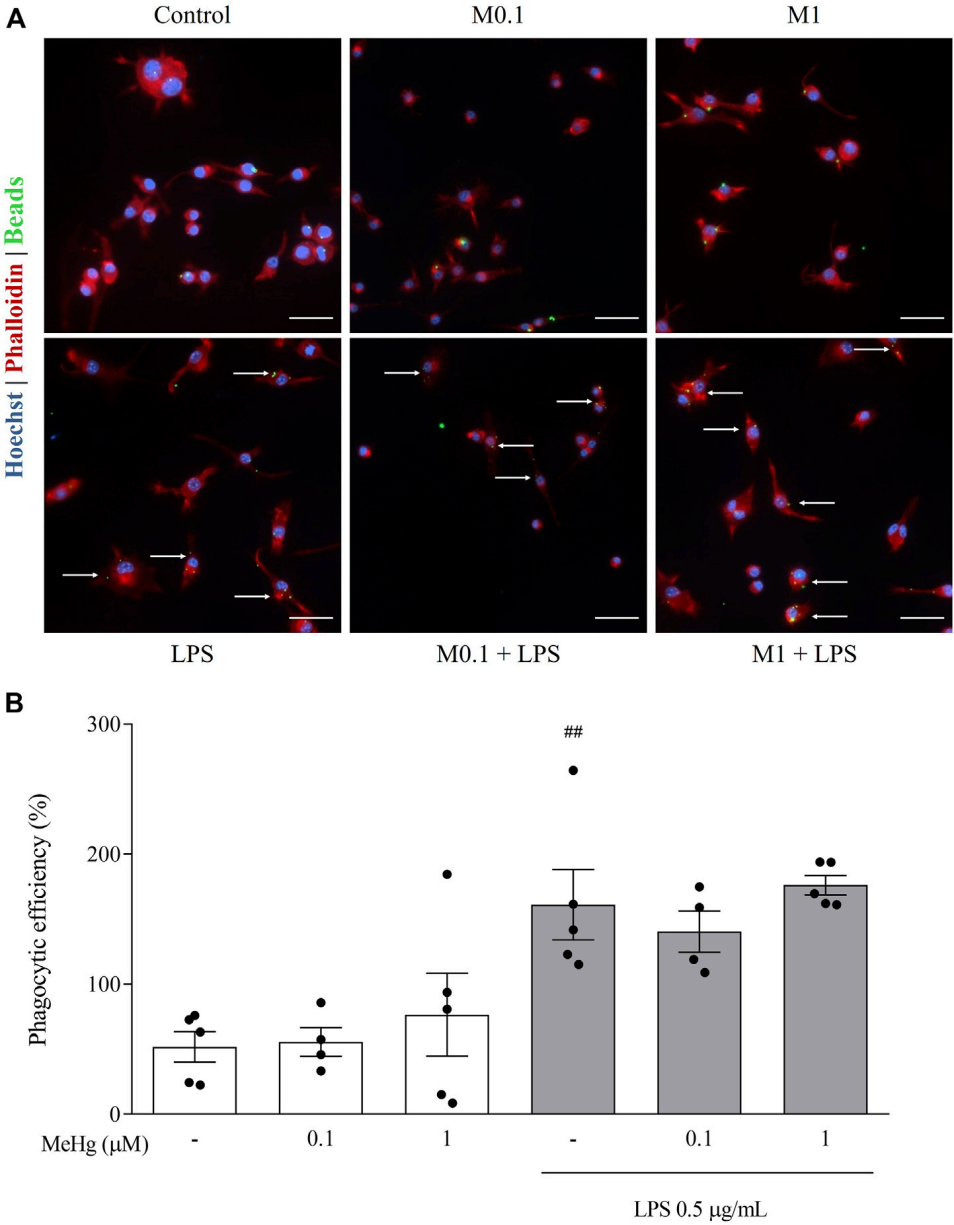
Effect of MeHg exposure (0.1, and 1 µM) on the phagocytic profile of BV-2 microglial cells exposed for 1 h and subsequently stimulated with LPS for 6 h, obtained through Phagocytic activity assay. **(A)** Representative images of BV-2 phagocytosis activity labelled with Hoechst 33342 (blue), Phalloidin (red) and fluorescent beads (green). Arrows show cells with internalized beads. Scale bars, 50 µm. **(B)** Assessment of phagocytic efficiency of BV-2 microglial cells. ##*p* < 0.01 vs. Control. Data are expressed as Mean ± SEM, *n* = 5. One-way ANOVA with Bonferroni’s multiple comparisons test.

To further study the impact of MeHg on the immune repertoire of microglia, we evaluated BV-2 microglial cells phagocytic activity using fluorescent latex beads uptake. Exposure to increasing concentrations of MeHg did not alter the phagocytic efficiency compared to control (*p* > 0.05, Figure 5). Additionally, the inflammatory activation of microglia with LPS strongly enhanced phagocytic uptake of beads (Control 51.8 ± 26.3% and LPS 161.1 ± 60.4%, *p* < 0.01). MeHg, up to 1 µM concentration did not affect the phagocytic capacity (*p* > 0.05). These results indicate that lower concentrations of MeHg do not affect the phagocytic efficiency of microglia, even in the presence of a pro-inflammatory booster.

## Discussion

MeHg is a potent neurotoxicant, which may induce severe toxic effects in both the developing and mature CNS (Yokoo et al., 2003). Microglial cells are the first line of CNS defence, modulating the local immune response (Shinozaki et al., 2019). Once exposed to an insult, microglia acquire an activated state which enables these cells to proliferate, change their morphology, migrate towards the injury site, and efficiently phagocyte pathogens (Thameem Dheen et al., 2007). Additionally, active microglia release pro-inflammatory mediators such as TNF-α, IL-1β, IL-6, NO, and ROS (Pinheiro et al., 2007; Shinozaki et al., 2019). The overactivation of microglia and neuroinflammation in the brain are thought to be key players in the aetiology and progression of neurodegenerative diseases, including Alzheimer’s disease, Parkinson’s disease, and multiple sclerosis (Yokoo et al., 2003; Aires et al., 2019). Previous studies have indicated that exposure to MeHg induces microglial activation, thus contributing to neurodegenerative processes (Thameem Dheen et al., 2007; Glass et al., 2010). Different stimuli are currently being used to model neuroinflammatory-driven neurodegenerative conditions, such as LPS, that could induce robust microglia activation (Wang-sheng et al., 2017). Despite the extensive characterization of neurotoxicity caused by MeHg exposure, the acute toxic effects caused by MeHg exposure in microglial cells are poorly understood.

MeHg-induced neurotoxicity is closely related to ROS generation (Chung et al., 2019). Since MeHg is a potent electrophilic agent, which allows it to react with thiol groups, a wide range of tissue damage occurs in response to its exposure. ROS can exert direct toxicity to cells, including DNA damage, protein function and activity changes, and a weak cellular antioxidant defence, leading to ROS accumulation and intracellular glutathione (GSH) depletion (Castoldi et al., 2000; Shenker et al., 2002). Among the CNS cells, astrocytes (Chang et al., 2019), neurons, and glia (Kaur et al., 2008) all have shown to be susceptible to the MeHg effect causing marked ROS generation.

Ni and others (2010) have reported increased ROS levels in a primary microglia culture already 1 min after 5 µM-MeHg treatment with raising levels until 10-min exposure. A MeHg dose-dependent increase in oxidative stress over time has been found to cause a significant reduction in intracellular GSH levels and subsequently induce microglia cell death (Ni et al., 2011). In another study, using a C6-glial cell line, 10 µM-MeHg induces a significant increase in ROS production at all the tested time points (30, 50, and 90 min). This effect was also observed when cells were exposed to 25 µM-MeHg, except for the 90 min time point, which decreased significantly the fluorescence intensity (Kaur et al., 2008). Moreover, the LPS treatment alone has been shown to significantly raise intracellular ROS production in HAPI microglial cells in a dose-dependent manner, starting from 1 to 10 ng/ml (Wang et al., 2004). In our study, we found a significant reduction in ROS production at 10 µM-MeHg concentration. Our results indicate that 10 µM-MeHg-challenged microglia, stimulated or not with LPS, reduces ROS formation, while at lower MeHg concentrations only a trend to modest variation is seen. Besides, upon LPS stimulation alone we observed an increase in ROS production, compared to the control group, as previously described (Wang et al., 2004). Similar results were reported, concerning the neurotoxic effect of MeHg in a study using SH-SY5Y-derived ρ^0^ cells exposed at concentrations of 1 and 10 μM-MeHg, for 30 min. Whereas the lowest concentration was reported to induce oxidative stress by increasing the fluorescence intensity, subsequently leading to cell death, 10 μM-MeHg did not provoke an increase in ROS production, indicating that, at this concentration, the predominant cell death pathway is not oxidative stress-dependent (Ishihara et al., 2016). Several hypotheses can explain the decreased fluorescence intensity at 10 µM-MeHg concentration, including a concentration and time-dependence and a weak cellular defence response, which together may lead to cell death. As previously described, ROS generation levels are influenced by MeHg concentration, as well as the exposure time (Kaur et al., 2008). At a concentration of 10 μM, MeHg has been found to induce 50% of neural cell death upon 1 day exposure, and to highly inhibit protein synthesis (Garg and Chang, 2006). Furthermore, several mechanisms are triggered when the cells are exposed to high MeHg concentrations. The combination of augmented ROS production (Chung et al., 2019) and intracellular GSH reduction makes cells more vulnerable, even to minor changes in intracellular ROS concentration when exposed over time, which may trigger the cell death signalling cascade (Ni et al., 2010). Thus, our results suggest that high MeHg concentration (10 μM), associated with extended time exposure, may induce a neurotoxic environment, which consequently leads to microglia cell death.

In the CNS, cytokines and chemokines are secreted mainly by glial cells, affecting the nature and intensity of immune responses (Ishihara et al., 2016). IL-6, TNF-α, and NO are active players in the immune process by inducing inflammation, which further leads to neurodegenerative disturbances (Pinheiro et al., 2007; Aires et al., 2019). Apart from their engagement to fight pathogens, the inflammatory response has been linked to the development of several neurodegenerative conditions, influenced by environmental exposure to toxicants such as MeHg (Garg and Chang, 2006; Cheng et al., 2021). Under physiological conditions, IL-6 and TNF-α levels are maintained at very low levels (Pinheiro et al., 2007; Ishihara et al., 2016). However, microglia stimulation can trigger the release of considerable amounts of pro-inflammatory mediators *in vitro*, which have already been correlated with CNS disease (Aires et al., 2019; Cheng et al., 2021), as well as observed under LPS stimulation (Sass et al., 2001). Our study shows the impact of MeHg on the production of two pro-inflammatory cytokines, IL-6 and TNF-α. BV-2 microglial cells were exposed to MeHg followed, or not, by stimulation with LPS, for 6 h. In the absence of LPS, microglial cells did not exhibit changes in the production and secretion of both pro-inflammatory cytokines when exposed to different MeHg concentrations. However, upon LPS stimulation, we observed a significant increase in IL-6 and TNF-α content, according to the previously observed in several studies (Pinheiro et al., 2007; Toyama et al., 2021; Yuan et al., 2021). Moreover, treatment with 10 µM-MeHg significantly decreased the LPS-stimulated cytokines production and release.

Several reports have highlighted discrepancies concerning IL-6 and TNF-α production and secretion during MeHg exposure in animal studies (Toyama et al., 2021) and microglial cell lines (Sass et al., 2001; Thameem Dheen et al., 2007; Ge et al., 2019). Indeed, in N9 microglial cells treated with several concentrations of MeHg for 8h, the results showed that 10 µM-MeHg induces a significant increase in IL-6 production (Garg and Chang, 2006). On the contrary, the MeHg exposure with the highest concentration (5 µM-MeHg) used in this study induced a low IL-6 production level measured in C6 glioma cells, while an intermediate concentration (2.5 µM) MeHg provoked an increase in IL-6 levels, on treated cells for 8 h and 16 h (Chang, 2007). Concerning TNF-α levels, different MeHg responses have also been reported. In microglial cells, following MeHg exposure, increasing TNF-α levels have been observed during chronic incubation (21 days at 100 nM), whereas at short exposure (1 day at 100 nM) its expression was barely perceptible (Shinozaki et al., 2019). Additionally, Tan and co-workers (2019) reported that at a low MeHg-concentration (2 ng/ml), for 48 h exposure, N9 murine microglial cells decrease TNF-α mRNA levels (Tan et al., 2019). Microglial primary culture stimulated with PAM (Ceccatelli et al., 2010), a toll-like receptor (TLR) ligand, have been found to reduce the secretions of IL-6, while maintaining unchanged TNF-α levels in a concentration-dependent manner (Bassett et al., 2012). Those contrasting findings reported by different authors may be explained through several differences in the experimental designs of the studies, namely MeHg concentrations, time of exposure, and cell origin. Nonetheless, our results, as well as earlier reports indicate that cytokine production may be concentration and time exposure-dependent.

Besides cytokine assessment, NO production pathway was also evaluated. In our study, we detected a higher iNOS expression on MeHg-treated cells, when challenged with LPS. However, with the highest concentration (10 µM) of MeHg, iNOS expression was considerably decreased. In line with these data, cells challenged with LPS significantly increased nitrates levels, while MeHg concentrations of 0.1–1 μM did not show significant differences. Additionally, cells exposed to 10 µM-MeHg, and subsequently with LPS, showed a marked reduction in nitrites levels. According to our results, earlier reports have also notified that LPS could induce a significant increase in iNOS mRNA expression and NO levels in rat primary microglia cells (Park et al., 2016). Besides, Tan and co-workers (2018) showed that a higher MeHg concentration exposure to N9 microglia cells leads to decreased mRNA expression of NO, whereas at lower concentrations the mRNA expression is increased, when exposed for 48 h (Fujimura et al., 2019). Their results, also confirmed that iNOS mRNA expression decreases with higher MeHg concentrations and its expression increases at low MeHg concentrations (Tan et al., 2019). Another study correlated the type of exposure with the iNOS detection. For acute exposure to 100 nM-MeHg, iNOS was barely detected and for chronic exposure at the same concentration, an increased signal was observed (Shinozaki et al., 2019). Thus, neurodegeneration induced by MeHg seems to be correlated with MeHg exposure in a concentration and time-dependent manner, consequently affecting the levels of production and secretion of pro-inflammatory factors, suggesting that the decreased contents of IL-6, TNF-α, NO and iNOS may be provoked by the cytotoxic effect of elevated concentrations, which leads to cell death.

The cytotoxicity effect induced by MeHg was determined by evaluating the metabolic activity, necrosis, and apoptotic cell death profile, following exposure to different MeHg concentrations, ranging from 0.01 to 10 μM, for 6 h. As expected, MeHg-induced cytotoxicity increased with the highest MeHg concentration tested (10 μM) in our study, by significantly decreasing the metabolic activity, while at lower concentrations only minor variations were observed. Accordingly, an earlier report has documented similar results. MeHg has been found to compromise the metabolic activity in NSC-34 cell culture, by significantly enhancing the resazurin reduction with increasing MeHg concentrations (up to 16 μM). Additionally, a trypan blue exclusion viability assay reported a significant concentration-dependent decrease in cell viability of cells exposed to MeHg for 48 h, reaching 0% of viability in cells exposed to 8 μM-MeHg (Chapman and Chan, 1999).

We then investigated a putative correlation between decreased metabolic activity and cell death, only using 1 and 10 µM-MeHg concentrations, since in the previous assay no significant differences were observed. Firstly, our results show increased PI uptake by BV-2 cells exposed to MeHg (10 µM), for 6 h, suggesting a necrotic-like cell death process. In contrast, at the lowest concentration tested, only a few cells, stimulated or not with LPS were found to be PI-positive. Recent data revealed that 3 µM MeHg-treated astrocytes, considerably increased PI uptake, suggesting a necrotic cell death, which was triggered by c-Jun N-terminal kinase (JNK)/MAPK signaling pathway (Pierozan et al., 2017). Interestingly, the mechanism by which MeHg seems to induce cell death is correlated with the intensity and the time exposure of this toxicant. Exposure to high MeHg concentrations (5 and 10 µM) for 1 h have shown to induce cerebellar granule cell death through necrosis, with simultaneous mitochondrial activity impairment, mitochondria de-energization, and plasma membrane lysis. Contrarily, lower MeHg concentrations were reported to maintain membrane integrity, mitochondrial function, and membrane potential, inducing cell death by apoptosis which coexisted with necrosis (20%). However, for a longer period, cells exhibited morphological characteristics of apoptotic cell death reaching the top apoptotic values after 18 h (Castoldi et al., 2000). Additionally, in HepG2 cells exposed to 1 µM-MeHg for 24 h, high levels of oxidative stress seemed to be induced, triggered by caspase-mediated apoptosis. Nevertheless, the predominant cell death mechanism was necrosis (Cuello et al., 2010).

As documented in primary cultures of cerebellar neurons, even MeHg low concentrations could trigger apoptosis, whereas necrosis induction may require higher MeHg concentrations (Kunimoto, 1994). Given that, in our study, microglia cells exhibited cell death through necrosis at high MeHg concentrations, we evaluated whether MeHg-driven cell death would occur by caspase activation even at low concentrations. We did not find differences between treatments in the proteolytic activity of caspase-3 and caspase-9, at 6 h MeHg exposure. It has been indicated in human HepG2 cells exposed to 2 mg/L MeHg that caspase-3 proteolytic activity increases after 8 h of exposure up to 24 h, while caspase-9 activity was enhanced after 2 h (Cuello et al., 2010). In primary cultured rat microglial cells, evidence suggests that MeHg induces the apoptotic pathway at 0.5 µM, reaching its maximum value 30 h after treatment (Nishioku et al., 2000). Not having observed any significant difference in our work, a potential explanation could be that the exposure time to MeHg did not allow the maximum values of caspase activation to be reached. Therefore, our data indicate that the predominant cell death pathway at high MeHg concentration seems to be necrosis, rather than apoptosis.

Phagocytosis is a central mechanism in host defence against invading pathogens and could be used as an immunotoxicity biomarker for environmental pollutant effects (Aroor and Baker, 1998; Fournier et al., 2000). Effective microglia phagocytosis is essential for the removal of pathogens and clearance of cell debris and apoptotic cells (Sierra et al., 2013), being a mechanism that has been well conserved. Thus, monitoring phagocytosis efficiency appears to be a good indicator for determining the immunotoxicity of contaminants in the environment (Fournier et al., 2000). Previous studies have shown a decrease in phagocytosis in different cells exposed to MeHg (Holloway et al., 2003; Schaafsma et al., 2015). In MeHg-challenged chicken white blood cells, the phagocytic function was significantly suppressed by increasing MeHg concentrations, ranging from 0.1 to 10 μg/ml. Noteworthy, cell viability was only affected above 1 μg/ml of MeHg (Holloway et al., 2003). Additionally, Schaafsma and others (2015) have shown that LPS-preconditioned microglia significantly increases phagocytic activity *in vitro* (Schaafsma et al., 2015). After assessing the metabolic activity, necrosis, and apoptotic cell death profile of BV-2 microglial cells upon MeHg exposure in our study, the neurotoxic effect of MeHg at high concentrations was highlighted, resulting in high rates of necrotic cell death. Based on these results, the highest 10 µM-MeHg concentration, which induced high rates of cell death, was not used on the phagocytic assays performed. Similar to previous results (Schaafsma et al., 2015), we observed a significant increase in phagocytic efficiency of microglial cells stimulated with LPS. However, we could not find a clear inhibitory effect in BV-2 cell phagocytic activity following submicromolar MeHg exposure.

In conclusion, our results support that acute exposure to high concentrations of MeHg reduces the metabolic activity of microglial cells and induces necrotic processes. Additionally, necrotic-like cell death seems to drastically reduce the production and secretion of several pro-inflammatory mediators on microglial cells. Further studies are needed to better dissect the complex molecular mechanisms underlying MeHg toxicity in microglial cells at acute and long-term chronic levels. The microglia-astrocyte interaction may also play a role in the MeHg-induced neurotoxicity in the brain and may be the focus of future studies.

## Data Availability

The original contributions presented in the study are included in the article/Supplementary Material, further inquiries can be directed to the corresponding author.
